# Type I CRISPR-Cas-mediated microbial gene editing and regulation

**DOI:** 10.3934/microbiol.2023040

**Published:** 2023-12-18

**Authors:** Zeling Xu, Shuzhen Chen, Weiyan Wu, Yongqi Wen, Huiluo Cao

**Affiliations:** 1 Guangdong Province Key Laboratory of Microbial Signals and Disease Control, Integrative Microbiology Research Centre, South China Agricultural University, Guangzhou 510642, China; 2 Department of Microbiology, Li Ka Shing Faculty of Medicine, The University of Hong Kong, Hong Kong

**Keywords:** CRISPR-Cas, Cascade, gene editing, gene repression, gene activation, anti-CRISPR

## Abstract

There are six major types of CRISPR-Cas systems that provide adaptive immunity in bacteria and archaea against invasive genetic elements. The discovery of CRISPR-Cas systems has revolutionized the field of genetics in many organisms. In the past few years, exploitations of the most abundant class 1 type I CRISPR-Cas systems have revealed their great potential and distinct advantages to achieve gene editing and regulation in diverse microorganisms in spite of their complicated structures. The widespread and diversified type I CRISPR-Cas systems are becoming increasingly attractive for the development of new biotechnological tools, especially in genetically recalcitrant microbial strains. In this review article, we comprehensively summarize recent advancements in microbial gene editing and regulation by utilizing type I CRISPR-Cas systems. Importantly, to expand the microbial host range of type I CRISPR-Cas-based applications, these structurally complicated systems have been improved as transferable gene-editing tools with efficient delivery methods for stable expression of CRISPR-Cas elements, as well as convenient gene-regulation tools with the prevention of DNA cleavage by obviating deletion or mutation of the Cas3 nuclease. We envision that type I CRISPR-Cas systems will largely expand the biotechnological toolbox for microbes with medical, environmental and industrial importance.

## 1. Introduction

Rapid developments in high-throughput sequencing technologies have deciphered genome sequences of a large number of isolated microorganisms with great medical, environmental and industrial importance, but functional characterization of specific genes and precise control of gene expression for metabolic engineering remain basically dependent on the availability of efficient genetic tools [1]. The discovery of prokaryotic adaptive immune systems named CRISPR-Cas (clustered regularly interspaced short palindromic repeats-CRISPR-associated proteins) systems, particularly the class 2 systems with single effector nucleases such as Cas9 and Cas12a, have attracted global interest for the development of novel genetic tools and have been widely used for gene editing and gene regulation not only in microorganisms but also in plants and animals [2]–[4]. Class 1 type I CRISPR-Cas systems are the most abundant CRISPR-Cas systems in nature and exhibit great potential for diverse biotechnological applications. Despite their complexity in structure, exploitations and applications of type I CRISPR-Cas systems in recent years have shown their advantages and importance in gene editing and gene regulation, particularly in genetically recalcitrant microbial strains [5]. In this review, we first briefly introduce the mechanisms of CRISPR-Cas adaptive immunity and their classifications with a focus on the type I CRISPR-Cas systems. Then, recent advancements in gene editing and gene regulation using the endogenous and heterologous type I CRISPR-Cas systems in different microorganisms are summarized and discussed.

## 2. CRISPR-Cas adaptive immune system

The CRISPR-Cas system, consisting of CRISPR arrays and *cas* genes, is a prokaryotic adaptive immune system that defends against the invasion of foreign genetic elements such as bacteriophages and plasmids [6],[7]. This adaptive immunity is composed of three stages: adaptation, crRNA expression and interference [8]. Generally, at the first adaptation stage, a segment of the invading DNA sequence is recognized and acquired by specific Cas proteins such as the Cas1 and Cas2 proteins. This segment is named as a protospacer and inserted into the CRISPR array to generate a new spacer following the duplication of the leader-proximal repeat sequence [9]. At the next crRNA expression stage, the whole CRISPR array is transcribed into a single precursor CRISPR RNA (pre-crRNA), which is further processed and sheared into small mature crRNA containing a spacer and parts of the flanking repeats by the host RNaseIII or a specific Cas protein with endoribonuclease activity such as the Cas6 protein [10],[11]. At the interference stage, crRNA and Cas proteins form an effector module, which is guided by the crRNA to recognize the protospacer in the invading bacteriophage or plasmid through complementary base paring [12]. In addition to crRNA, the presence of a protospacer adjacent motif (PAM), which is a 2- to 5-bp motif located immediately upstream or downstream of the protospacer for discrimination between “self” and “non-self” sequences, is normally required for specific protospacer recognition [13]. Finally, the invading bacteriophage or plasmid is cleaved and degraded by a Cas nuclease which is either a part of the effector module or an additional single Cas protein [14]–[16].

## 3. Classification of CRISPR-Cas systems

**Figure 1.**
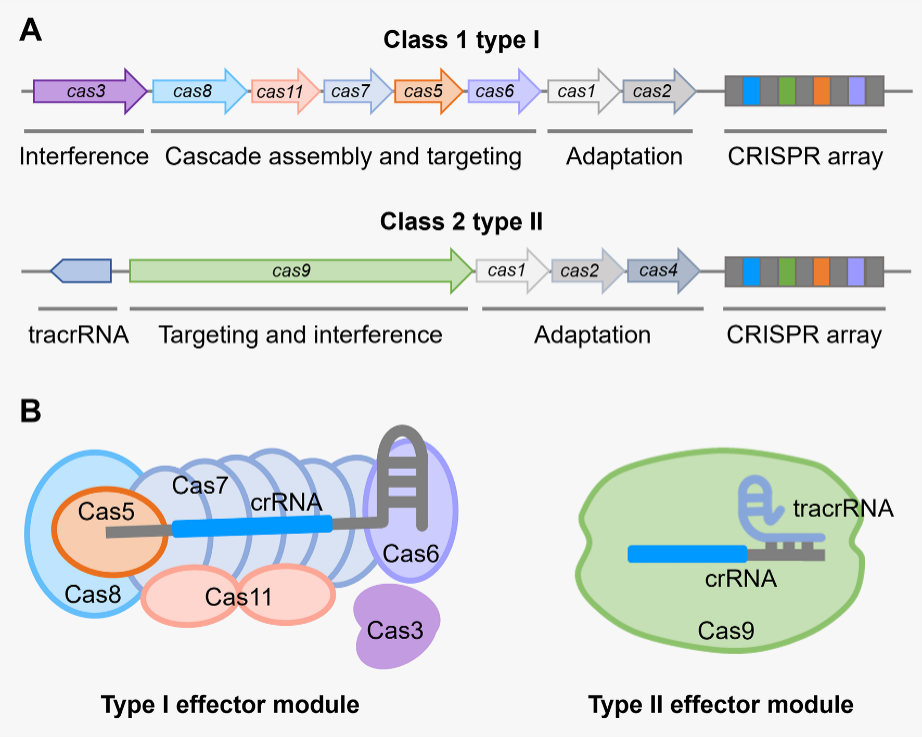
Comparison of class 1 type I and class 2 type II CRISPR-Cas systems. (A) CRISPR-*cas* loci of the type I and type II systems (type I-E and type II-A systems are shown as examples). Type I systems use a Cascade complex, which contains multiple different Cas proteins, for DNA targeting and interference, while type II systems use a single large protein for DNA targeting and interference. (B) Schematics of type I-E effector module and type II-A effector module. The type I-E effector module contains a Cas3 protein and a crRNA-bound Cascade. The type II-A effector module contains a Cas9 protein and a fusion of crRNA and tracrRNA.

CRISPR-Cas systems are found broadly distributed in around 40% of bacterial genomes and more than 85% of archaeal genomes and display remarkable diversity regarding compositions and sequences of Cas proteins, architectures of genomic loci, *etc*. [17]. Based on the rapid progress in the discovery of CRISPR-Cas systems with expanding genomic and metagenomic information, the classification of CRISPR-Cas systems is updated periodically [17]–[21]. Currently, CRISPR-Cas systems are classified into two main classes (1 and 2), six types (I to VI) and at least 33 subtypes. Class 1 systems utilize a crRNA-binding effector complex such as the Cascade (CRISPR-associated complex for antiviral defense), which is composed of a crRNA and multiple Cas proteins to recognize and interfere with invading bacteriophage genomes or plasmids in the class 1 type I system (Figure 1). Class 2 systems use a large multidomain crRNA-binding protein, such as Cas9 in the class 2 type II system, to execute the same functions of nucleic acid recognition and degradation as the effector complex in class 1 systems (Figure 1).

## 4. Type I CRISPR-Cas systems

Owing to the simplicity of class 2 CRISPR-Cas systems, which employ a single Cas effector protein for nucleic acids targeting, these systems have been extensively exploited and developed for gene editing, gene regulation and many other applications in diverse organisms [22]–[24]. Noticeably, the class 1 type I CRISPR-Cas systems are the most widespread systems in nature, and they also provide tremendous opportunities for promising applications in spite of their structural complexity [25]. Type I CRISPR-Cas systems feature the signature protein Cas3, which is a large protein consisting of both helicase and nuclease domains and is responsible for target DNA cleavage [26].

There are two main modules in the type I systems. One is the conserved adaptation module, which is generally composed of core proteins Cas1 and Cas2, and the other one is the effector module, consisting of a Cascade effector complex and the signature nuclease Cas3 [27]–[29]. In general, the Cascade contains four Cas proteins, i.e., Cas5, Cas6, Cas7 and Cas8 [20]. The Cas6 protein therein is not only a component of the Cascade but also an endoribonuclease responsible for crRNA processing. After its cleavage at the repeat sequence in the pre-crRNA to yield mature crRNAs, Cas6 stays bound to the 3′ hairpin structure of the mature crRNA and then recruits other Cas proteins to form a Cascade-crRNA complex [30],[31]. The Cascade-crRNA complex then conducts RNA-guided DNA targeting. If a PAM sequence is present, the Cascade recognizes the target DNA by base pairing between the target strand and the crRNA [32],[33]. Binding of the crRNA and the target strand sequence leads to the formation of an R-loop at the target locus [12],[33]. In this process, base pairing of the spacer in the crRNA and the target DNA between the seed sequence, which is usually the first eight nucleotides proximal to the PAM, is essential for the recognition and targeting of the Cascade complex [34]. Subsequently, the enzyme Cas3 is specifically recruited to the Cascade/R-loop and progressively degrades the non-target DNA strand [26],[28].

**Figure 2.**
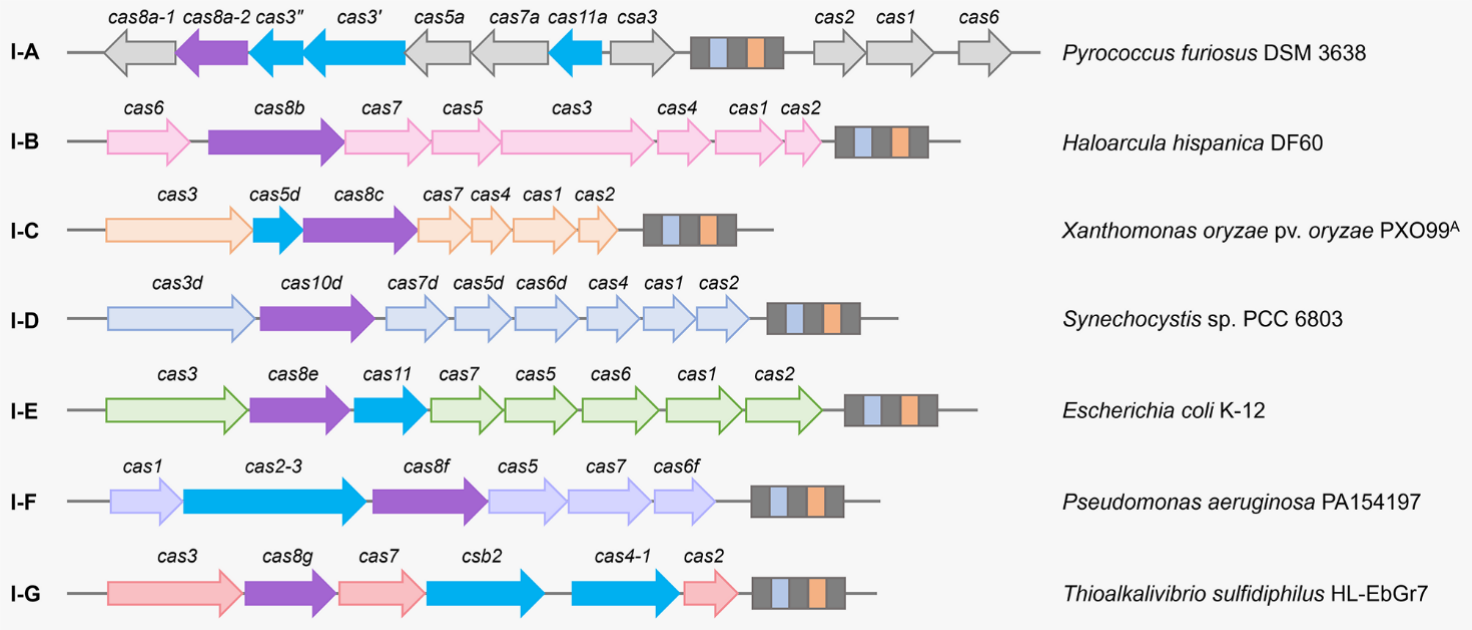
Representative CRISPR-*cas* loci for different subtypes of the type I CRISPR-Cas system. Genes used for defining different subtypes are highlighted in purple. Some unique genes or genes with unique features in some subtypes, such as *cas3*′, *cas3*″ and *cas11a* in the type I-A system, *cas5d* in the type I-C system, *cas11* in the type I-E system, *cas2-3* fusion in the type I-F system and *cas5-6* (*csb2*) and *cas4-1* fusions in the type I-G system, are highlighted in blue.

Subtypes of type I CRISPR-Cas systems are classified according to the different genetic arrangements and unique *cas* genes as well as repeat sequences and their lengths. Because the Cas8 proteins showed no detectable similarity between different subtypes of the type I systems, they have been selected to serve as the subtype signature proteins [18]. Type I systems were initially divided into seven subtypes from I-A to I-F and I-U, where U stands for uncharacterized functions of the signature protein (Figure 2) [19]. Along with the deciphering of the composition, structure and function of the Cascade effector in recent years, the I-U system has been reclassified as the I-G system [17],[35],[36]. Noticeably, some derived variants of type I-B, I-D and I-F systems were discovered to be encoded by Tn7-like transposons which lack the Cas3 nuclease for interference [37]–[39].

## 5. Type I CRISPR-Cas-mediated gene editing

### 5.1. Gene editing using endogenous type I systems

**Figure 3.**
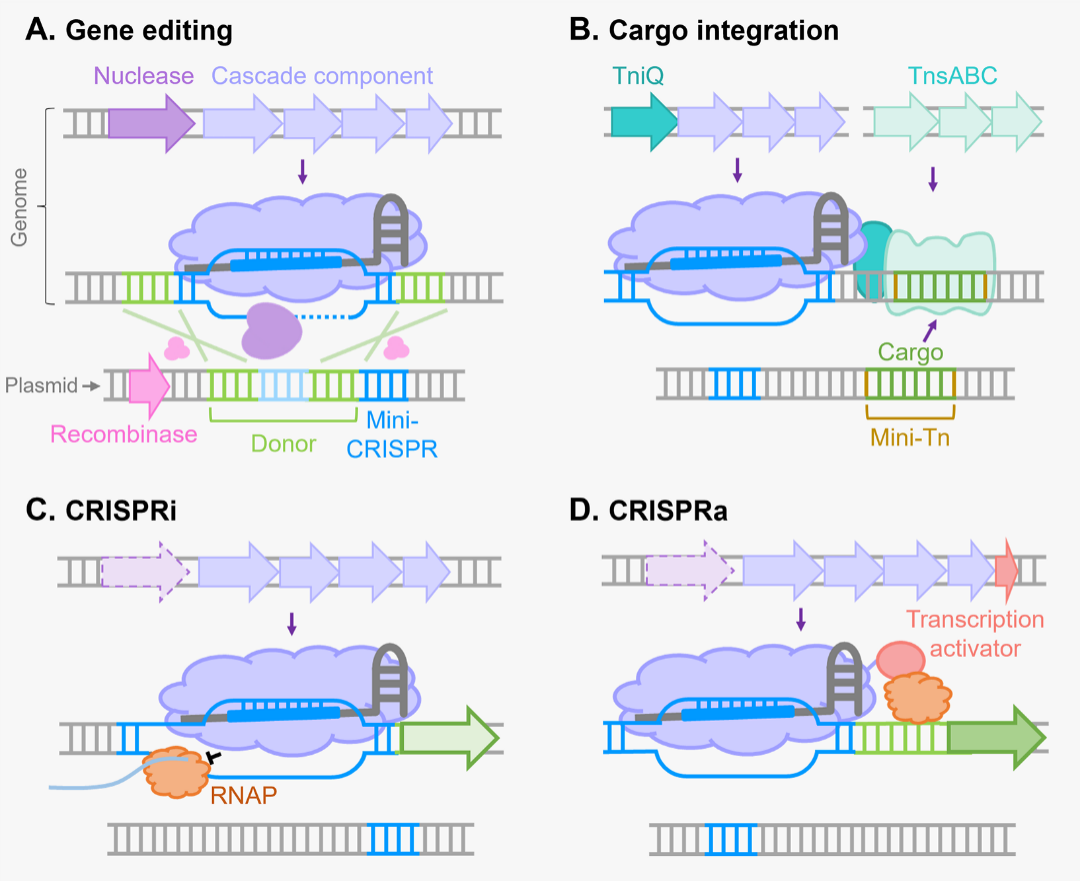
General strategies to achieve microbial gene editing and regulation using type I CRISPR-Cas systems. (A) A diagram showing the endogenous type I CRISPR-Cas-mediated gene editing. A mini-CRISPR expressing the crRNA to guide the Cascade to target genomic locus and a donor sequence for homology-directed DNA repair are provided for precise gene editing. Precise editing such as gene deletion, gene insertion and single base substitutions can be achieved by proper design of the donor sequence. Recombinases are sometimes required to improve the frequency of homologous recombination and thus increase the efficiency of gene editing. (B) Programmable genomic integration using the type I-F3 CAST. A TniQ-Cascade complex and transposition proteins TnsA, TnsB and TnsC are guided by the crRNA to the target genomic locus, which enables the integration of the mini-transposon (Mini-Tn) ~50 bp downstream of the target site. (C) Type I CRISPR-Cas-mediated CRISPRi. After deleting or mutating the Cas3 nuclease to disrupt the DNA cleavage function of the type I CRISPR-Cas system, only a mini-CRISPR is required to express a crRNA which guides the Cascade complex to a target genomic locus (preferably the promoter region) for downstream gene repression by preventing the recruitment or movement of RNA polymerase (RNAP). (D) Fusing a transcription activator to a Cascade component can convert the CRISPRi system to a CRISPRa system for downstream gene activation.

In recent years, increasing studies have explored the potential of the abundant type I CRISPR-Cas systems for gene editing and shown great success in manipulating not only prokaryotic genomes but also genomes of eukaryotic cells, including plant and human cells [40]–[45]. Although type I systems are structurally complicated, their presence in a majority of bacterial and archaeal strains enables them to act as endogenous gene editors to manipulate their own genomes [46]. Therefore, repurposing the endogenous type I CRISPR-Cas systems for gene editing is convenient, and it simply requires a single vector to express a crRNA that can guide the Cascade and Cas nuclease to desired genomic targets and carry a donor sequence for homology-directed DNA repair (Figure 3A) [46],[47]. However, CRISPR-Cas systems are highly diverse in different hosts. For instance, even systems belonging to the same subtype may recognize different PAM sequences for DNA targeting in different bacterial strains [16],[48],[49]. Therefore, if a type I CRISPR-Cas system is present in a strain, it is necessary to first characterize the system comprehensively to clarify all elements that are required for DNA targeting and interference, including PAM sequence, repeat sequence and spacer length, by a combination of bioinformatic predictions and plasmid or genome interference assays, as described previously [46],[47],[50]. Here, we move to introduce some examples that employed endogenous type I systems to achieve efficient and diversified gene editing in multiple bacterial and archaeal strains with industrial and clinical significance.

#### 5.1.1. Endogenous type I-A system

The type I-A system in *Sulfolobus islandicus* is the first example that showed the success of the type I system for precise gene editing, exhibiting active genome targeting when a plasmid is introduced to express a crRNA targeting the *lacS* gene and then exhibiting efficient deletion of the target sequence with simultaneous provision of the donor template in the plasmid [51]. In addition, gene editing based on an endogenous type I-A system was also explored in Gram-positive bacterial species *Clostridium saccharoperbutylacetonicum* and *Heliobacterium modesticaldum*. In *C. saccharoperbutylacetonicum*, two plasmids carrying a donor template and a crRNA-expressing element, respectively, were successively transformed into the cell to achieve various types of gene editing, such as single base substitution, gene deletion and insertion [52]. Due to the absence of the Cas8a protein, the PAM sequence for the type I-A system in *H. modesticaldum* was re-evaluated and determined as “5′-NNRCBN-3′” instead of “5′-CCN-3′” as found in *S. islandicus* and *C. saccharoperbutylacetonicum* [53]. Compared to the conventional gene editing method which was based on the non-replicating vector and showed extremely low efficiency (less than 1%), gene disruption by replacement based on the endogenous type I-A system was achieved with more than 80% efficiency by introducing a single editing plasmid into *H. modesticaldum* [53].

#### 5.1.2. Endogenous type I-B system

The genus *Clostridium* consists of important industrial strains that produce valuable chemicals as well as notorious pathogens causing global health threats [54]. Although genome-editing tools based on Cas9 and Cas12a were established for some *Clostridium* species, they frequently exhibit toxic effects to bacterial cells [55]. The type I-B system is the most abundant CRISPR-Cas system present in nature as well as the most abundant system in *Clostridium* [56]. In addition to the I-A system, endogenous type I-B systems are intensively explored in *Clostridium* species to overcome the difficulties of conventional gene editing methods. The first demonstration of the endogenous type I-B system in *Clostridium* for gene editing was reported in *C. pasteurianum*, a microorganism with biotechnological potential for the conversion of waste glycerol to butanol [57]. Introduction of a synthetic crRNA-expressing element and a donor template yielded 100% efficiency of gene deletion in *C. pasteurianum* based on the endogenous type I-B system, while the heterologous Cas9 system only generated 25% of the total yield of edited cells [56]. Gene editing efficiency comparisons between the endogenous type I-B system and the heterologous Cas9 system were also performed for *C. thermocellum*, an important industrial biocatalyst with robust lignocellulose-solubilizing activity [58], and *C. butyricum*, a widely used probiotic in humans and food animals [59],[60]. In *C. thermocellum*, it was shown that the type I-B system yielded 40% gene editing efficiency, while the Cas9 system yielded 12.5% gene editing efficiency at the same genomic locus [61]. In *C. butyricum*, expression of Cas9 resulted in extremely poor transformation, owing to its toxicity, while the endogenous type I-B system dramatically increased the plasmid delivery rate and successfully deleted genes with 100% efficiency [62]. Moreover, gene editing using endogenous type I-B systems was also demonstrated in multiple other bacterial or archaeal species such as *C. tyrobutyricum* [63], *C. difficile* [64], the industrial biofuel-producing microorganism *Thermoanaerobacterium aotearoense* [65] and the polyploid haloarchaeon *Haloarcula hispanica* [66],[67].

#### 5.1.3. Endogenous type I-C system

Unlike industrial strains and human pathogens, CRISPR-Cas systems in phytopathogenic bacteria have not been well characterized and developed for biotechnological applications. *Xanthomonas oryzae* pv. *oryzae* (Xoo) is a causal agent of rice bacterial blight and a major threat to rice production [68]. Genome analysis and self-targeting assay revealed that Xoo PXO99^A^ contains a functional type I-C system in its genome [69]. After thorough characterization of the PAM sequence, repeat sequence, spacer length and activity of the I-C system in this strain, Jiang and colleagues constructed a plasmid carrying a crRNA-expressing element, a donor template and an additional phage-derived recombination system and then utilized this plasmid to efficiently achieve various types of gene editing, including gene deletion, gene insertion, single base substitution and gene replacement [70]. Moreover, they demonstrated that the type I-C system cleaves target DNA unidirectionally, and the system enables the generation of large genomic deletions up to 212 kb with high efficiency [70].

#### 5.1.4. Endogenous type I-D system

The type I-D system is prevalent in cyanobacteria and archaea [71],[72]. Unlike other type I systems, the subtype signature protein of the type I-D system is not a Cas8 homologous protein but Cas10d, which is a type III Cas10-like large subunit (Figure 2) [73]. The Cas10d protein was demonstrated as a functional counterpart of Cas8 for PAM recognition and stabilization [42]. Using a self-targeting assessment assay, Bost et al. verified the activity of the type I-D system in *Sulfolobus acidocaldarius* and determined that the PAM sequences of this system are 5′-CCA-3′, 5′-GTA-3′ and 5′-TCA-3′ [74]. Deletion of *upsE* and single base substitution in *upsE*, a gene encoding the UV pili assembly ATPase, were next achieved in *S. acidocaldarius* with the provision of different donor templates. Gene editing based on the endogenous type I-D system offered a significant improvement in both efficiency and speed compared to the existing method [74].

#### 5.1.5. Endogenous type I-E system

The type I-E system is the most well-studied type I system, while few endogenous type I-E systems have been harnessed for bacterial gene editing. *Lactobacillus crispatus* is one of the predominant species in the human vaginal microbiome and plays an important role in poultry intestinal health [75],[76]. Despite the importance of this species in preventing infectious diseases, little is known about its genetic basis, owing to its genetic recalcitrance. Based on the type I-E system in *L. crispatus*, flexible and efficient gene editing, including gene deletion and insertion and single base substitution, was achieved [49].

#### 5.1.6. Endogenous type I-F system

The type I-F system was first shown to generate large deletion mutations, such as the remodeling or deletion of entire pathogenicity islands in *Pectobacterium atrosepticum*, when it is reprogrammed to target the dispensable genomic islands without the provision of a donor template [77]. Similarly, the endogenous type I-F system in *Serratia* sp. ATCC 39006 was designed to target the *flhC* gene by introducing a plasmid-encoded crRNA, which caused cell death and enabled the selection of *flhC* mutants that escape targeting [78]. When a donor template was provided simultaneously with the crRNA-expressing element, type I-F systems were demonstrated to be capable of precisely editing genomes of *Pseudomonas aeruginosa* and *Zymomonas mobilis*, an opportunistic human pathogen and an important industrial microorganism, respectively [79],[80]. In *Z. mobilis*, gene deletion and insertion and single base substitution can be realized with 100% efficiency, but deletion of large genomic fragments such as a 10-kb fragment was only achieved with less than 50% efficiency [80]. To increase the deletion efficiency for large genomic fragments, a recent study created an nCas3 variant by replacing the catalytic residues with alanine and showed that the I-F Cascade-nCas3 enabled gene insertion, gene deletion, single base substitution and deletion of one or even two large genomic fragments at once with almost 100% efficiency [81].

#### 5.1.7. Endogenous type I-G system

Type I-G systems contain fusions of Cas4-Cas1 and Cas5-Cas6 (Figure 2). Cas5-Cas6 is known as Csb2, which acts as the Cas6 protein to process pre-crRNA and remains bound to the 3′ end of the mature crRNA after processing [36]. Gene editing using the type I-G system was demonstrated in the genetically recalcitrant strain *Bifidobacterium animalis* subsp. *Lactis*, which has a variety of potential health benefits [82]. By introducing a crRNA targeting the 5′ end of the tetracycline resistance gene *tetW* as well as two 600-bp flanking homologous arms into *B. lactis*, Pan et al. successfully obtained a 500-bp deletion which included the promoter sequence, the start codon and a portion of the 5′ end of the *tetW* gene, restoring the sensitivity of the strain to tetracycline [82].

### 5.2. Gene editing using heterologous type I systems

Although a great number of studies have shown the feasibility, convenience and efficiency of repurposing the endogenous type I systems for gene editing in their hosts, whether type I systems can be expressed and stay active in heterologous hosts is poorly explored. With the accumulating well-characterized type I systems and their greater capability and efficiency in gene editing than the Cas9 and Cas12a systems, as demonstrated in some bacterial hosts, it is desirable to expand the application range of type I systems for heterologous gene editing. Due to the complicated structural composition of type I systems, efficient delivery and stable expression of the type I CRISPR-Cas components in target cells are a precondition for widespread applications of these systems. Here, we introduce some examples that harnessed the type I-C, type I-F (and its variant I-F3) and type I-G systems for gene editing in heterogenous bacterial cells.

#### 5.2.1. Heterologous type I-C system

The type I-C system was the first type I system optimized for heterologous gene editing in bacteria. Compared to other type I systems, the type I-C system is streamlined, with only four Cas proteins. Three Cas proteins, Cas5, Cas7 and Cas8, assemble the Cascade to recruit the nuclease Cas3. To stably express these type I-C Cas proteins, a previous study employed an integrative, isopropyl β-D-1-thiogalactopyranoside (IPTG)-inducible pUC18T-mini-Tn7T-Lac plasmid to carry four type I-C effector *cas* genes (*cas3*-*cas5*-*cas8*-*cas7*) from a *P. aeruginosa* isolate and then inserted the *cas* genes into the PAO1 genome to generate a PAO1^IC^ strain, which enables the expression of heterologous *cas* genes from the genome [83]. Introduction of crRNA-expressing plasmids to target the *phzM* gene led to deletions of 23.5, 52.8 and 60.1 kb large genomic fragments at the target site [84]. Noticeably, introducing mutated nucleotides in the repeat sequence of the CRISPR array to disrupt homology between these sequences greatly increased the editing efficiency to 94~100% [84]. Importantly, large genomic deletions than 1 kb obtained by the type I-C system occupied 98.6% of the assayed survivor cells, while only 5.6% were obtained by the type II Cas9 system, which indicated the greater capacity of the type I-C system to generate deletions of large genomic fragments than the Cas9 system [84]. An “all-in-one” vector which carries the crRNA-expressing element and the *cas3*, *cas5*, *cas8*, *cas7* genes was further constructed to enable the expression of the type I-C system and efficient gene editing in other hosts such as *Escherichia coli*, *Pseudomonas syringae* and *Klebsiella pneumoniae* [84].

#### 5.2.2. Heterologous type I-F and I-F3 systems

Similarly, another integrative mini-CTX-*lacZ* plasmid was utilized to carry the entire type I-F *cas* operon, which is composed of *cas1*, *cas2-3*, *cas8f*, *cas5*, *cas7* and *cas6* genes from a clinical *P. aeruginosa* isolate PA154197 [85]. Integration of the type I-F *cas* operon into multiple *Pseudomonas* strains, including the PAO1 strain and other clinical *P. aeruginosa* isolates as well as a *P. putida* strain, enables the stable expression of *cas* genes from their genomes. In some clinical strains, the type I-F system showed significantly higher targeting efficiency compared to the Cas9 system, which highlighted that the type I-F system might be a superior system for gene editing in clinical strains [85]. The integrated system in these strains is further harnessed as an “endogenous” type I-F system to achieve various types of gene editing, such as gene deletion and insertion and single base substitution, by introducing a single plasmid carrying a crRNA-expressing element and a donor template [85].

CRISPR-associated transposons (CAST), some CRISPR-Cas systems which are nuclease-deficient and encoded by Tn7-like transposons, were discovered in recent years. These systems are shown to execute RNA-guided DNA transposition and therefore are promising for programmable, highly efficient and site-specific integration of DNA fragments (cargo), as this type of genome modification does not require DNA cleavage and homology-directed repair involving endogenous or heterologous repair machinery (Figure 3B) [86]. Type I-F3 CAST, consisting of a Cascade and TniQ (homolog of TnsD in the prototypical Tn7 transposon) which complexes with the Cascade and facilitates interaction between the Cascade and the transposition machinery TnsABC [87], is the largest group of sequenced CAST elements and has been repurposed for gene editing in heterologous hosts and strains in the microbial communities [88]–[90]. For example, DNA integration at ~50 bp downstream of the genomic site targeted by the crRNA was achieved in an *E. coli* host by using a type I-F3 CAST from *Vibrio cholerae* [86].

#### 5.2.3. Heterologous type I-G systems

A type I-G system from *Thioalkalivibrio sulfidiphilus* was recently employed to manipulate the *E. coli* genome [91]. The type I-G operon, containing *cas3*, *cas8g*, *cas7* and *csb2*, and a mini-CRISPR targeting the *lacZ* gene were introduced into the *E. coli* MG1655 strain with a two-plasmid system, which yielded long-range bidirectional deletion of the *E. coli* genome likely through error-prone end joining pathways. When a donor template was further provided, the type I-G system generated precise deletion of a 13-kb sequence in the genome but with low efficiency (20%). Notably, when the Cas3 protein was replaced with its variant Cas3 K39A, which lacks helicase activity, the efficiency of targeted 13-kb deletion was increased to 90%, suggesting that a single nick in the target site might promote homology-directed DNA repair [91].

## 6. Type I CRISPR-Cas-mediated gene regulation

CRISPR-Cas-based gene editing in prokaryotes is mainly dependent on the homology-directed recombination between donor template and genomic DNA. Some studies have shown the incorporation of exogenous recombinases such as the phage λ-red recombination system can effectively elevate the editing efficiency of CRISPR-Cas systems. However, a majority of prokaryotes have extremely poor recombination frequencies, and whether exogenous recombinases remain active in a large number of prokaryotic cells is not sure. Thus, instead of the modification of genome sequences, alternative strategies to easily control the expression of target genes using CRISPR-Cas systems were also explored, triggering the development of CRISPR interference (CRISPRi) for gene repression and CRISPR activation (CRISPRa) for gene upregulation [92]–[94]. In principle, type I CRISPRi works by inactivating the Cas3 nuclease or preventing it from approaching the R-loop which is formed by the crRNA-guided binding of the Cascade and the protospacer sequence. As a result, stable binding of the Cascade at specific genomic sites prevents the recruitment of transcription factors and RNA polymerase (RNAP) or blocks the movement of RNAP (Figure 3C). The major difference between CRISPRa and CRISPRi is that Cas subunits of the Cascade in the CRISPRa system are fused with transcriptional activators, which recruits the RNAP to the promoter region of target genes and thus enhances genes expression (Figure 3D). So far, type I-E systems in *E. coli* and *Gluconobacter oxydans*, type I-B systems in *H. hispanica* and *Haloferax volcanii* and type I-F systems in *P. aeruginosa* and *Z. mobilis* have been engineered to achieve targeted modulation of gene expression.

### 6.1. Gene regulation with Cas nuclease-null type I systems

Unlike the applications for gene editing, the type I-E system was intensively explored for gene regulation. In *E. coli*, deletion of the *cas3* gene converted the type I-E system into a programmable CRISPRi system [95],[96]. It was shown that crRNA-guided Cascade targeting the promoter regions yielded the strongest repression, while targeting the open reading frame (ORF) sequence reduced the repression intensity [95],[96]. As crRNAs in type I systems can be transcribed from a single CRISPR array, it was demonstrated that the type I-E CRISPRi system was able to simultaneously repress multiple genes with a CRISPR array containing multiple spacers [95],[96]. However, the placement of spacers within the array was shown to affect the intensity of gene repression, with the most potent repression effect at the first position [95]. Moreover, it was shown that the type I-E CRISPRi system is applicable in diverse *E. coli* strains and even *Salmonella typhimurium* by transferring the Cascade and crRNA-expressing elements into *S. typhimurium* [95],[96]. After the type I-E CRISPRi system was established in *E. coli*, its application for metabolic engineering was evaluated. It was shown that repression of the *gltA* gene led to an elevation of acetate production in *E. coli* [97]. By further introducing the poly-3-hydroxbutyrate (PHB) biosynthetic pathway, acetate accumulation in the strain was eliminated, but PHB production could be increased [97]. Screening based on a combinatorial gene expression library, the endogenous type I-E CRISPRi system in *E. coli* enabled the identification of a set of variants that exhibited significant increases in malonyl-CoA flux and up to a 98% increase of the 3-hydroxypropionate production [98]. In addition to *E. coli*, a type I-E CRISPRi system was also developed in a *G. oxydans* strain, which contains a type I-E system, with the Cas3 protein naturally inactivated. Using this CRISPRi system, important roles of the pentose phosphate pathway and the Entner-Doudoroff pathway in the carbon center metabolism were identified [99]. These studies demonstrated that the type I-E CRISPRi system is an easy and effective method to regulate internal metabolic pathways.

When single effector proteins such as Cas9 and Cas12a were utilized to develop gene repression tools in the halophilic archaeon *H. volcanii*, it was found that only very small amounts of them were solubly expressed, possibly owing to the high salt concentration in the cell [100]. Because many archaea have type I-B systems in their genome, and the type I-B system is the best-characterized system in *H. volcanii*, this system was developed as an endogenous CRISPRi tool. It was noticed that the Cas6b protein is dispensable for the binding of Cascade to the target sequence [101]. Therefore, the *cas6b* gene was also deleted in *H. volcanii* in addition to the deletion of *cas3* to repurpose the system for gene repression, and a Cas6b-independent method which was alternatively dependent on the two tRNA processing enzymes RNase P and tRNase Z was used for easier crRNA maturation [100],[102].

The type I-F system is another popular system explored for endogenous and heterologous gene regulation. Deletion of the *cas2-3* gene in *Z. mobilis* enables the endogenous type I-F system for targeted gene repression [80]. Consistent with the repression effect of the type I-E CRISPRi system, crRNA-guided targeting at the promoter region led to a robust repression of the target reporter gene *mCherry*, which decreased the fluorescence intensity by around 88%. Meanwhile, targeting the ORF region decreased the fluorescence intensity by 23~75% [80]. It was demonstrated that combination of the type I-F *cas* operon (Δ*cas2-3*) and a crRNA-expressing element is transferable to repress target genes in different *P. aeruginosa* strains using the mini-CTX-*lacZ* plasmid [85]. In addition, the type I-F system from *P. aeruginosa* was also repurposed to achieve gene activation by fusing the activator RpoD to the Cas7 protein which functions as several copies to bind the protospacer in the Cascade. As tested in the model electroactive microorganism *Shewanella oneidensis*, crRNA-guided Cascade targeting the prioritized loci upstream of the transcription start site successfully activated the downstream gene expression [103]. This system was also demonstrated as a dual-regulation tool since it can be used to repress gene expression by binding with ORF regions of target genes as well [103].

### 6.2. Gene regulation with endogenously active Cas nucleases

**Figure 4.**
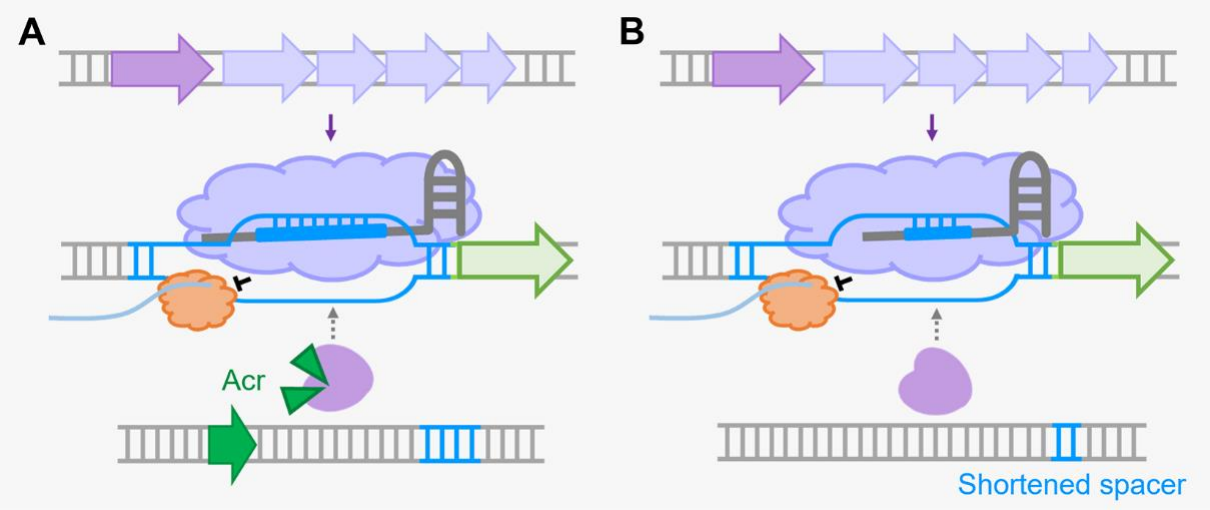
Repurposing the type I CRISPR-Cas system for gene regulation without pre-deletion or mutation of the Cas nuclease. (A) Simultaneous expression of crRNA with an anti-CRISPR protein (Acr) to inactivate the Cas3 nuclease enables desired gene repression in a microbial host which encodes an active endogenous type I CRISPR-Cas system. (B) When the spacer in the mini-CRISPR is shortened, the crRNA-Cascade complex can still bind to the target genomic locus but loses its ability to recruit Cas3 nuclease for DNA cleavage. Binding of Cascade at the target genomic site prevents the recruitment or movement of RNAP, which leads to the repression of downstream genes.

As type I systems are widespread in bacterial strains, the presence of the Cas3 nuclease (Cas2-3 in the type I-F system) frequently prevents CRISPRi applications. In the past few years, a group of CRISPR-Cas antagonists named anti-CRISPR proteins (Acr) were discovered in mobile genetic elements, functioning to inhibit CRISPR-Cas immunity [104],[105]. For example, AcrIE1 was found to inhibit Cas3 activity, and the use of AcrIE1 converted the endogenous type I-E system into a programmable CRISPRi system (Figure 4A) [106]. Regarding the type I-F system, there are 24 Acr proteins identified so far; and two of them, namely, AcrIF3 and AcrIF23, have been known to prevent the recruitment of Cas2-3 nuclease to the targeting site and inhibit the nuclease activity of Cas2-3, respectively [107],[108]. In a recent study, a new type I-F CRISPRi system with the simultaneous expression of the *cas* operon and the *acrIF3* or *acrIF23* gene was developed, which enables the type I-F CRISPRi system to work in a broad range of hosts, including strains expressing active endogenous type I-F systems [109]. By using this new type I-F CRISPRi system, novel regulatory mechanisms of antibiotic resistance were revealed conveniently in *P. aeruginosa*.

It was shown that spacer length could affect CRISPR-Cas functionality such as DNA interference and adaptation [110],[111]. Therefore, Du et al. systematically examined the effect of spacer lengths on CRISPR-Cas-mediated DNA cleavage and adaptation in *H. hispanica*, which revealed that CRISPR-Cas functionalities including DNA interference and adaptation are significantly reduced with the decreasing length of spacers [66]. Interestingly, it was found that target gene expression could be significantly inhibited by the endogenous type I-B system even with an active Cas3 nuclease when the spacer length was reduced to 24 bp (Figure 4B). By expressing crRNAs with different spacer lengths to target different genes, the endogenous type I-B system was designed to simultaneously achieve desired gene repression and gene editing [66].

## 7. Advantages of type I CRISPR-Cas gene editing and regulation

The emergence and utility of CRISPR-Cas systems have revolutionized biotechnologies including gene editing and gene regulation, which are indispensable tools for biological research. With the identification and characterization of increasing type I CRISPR-Cas systems in bacteria and archaea, repurposing these naturally abundant type I systems for gene editing and gene regulation is becoming more and more common owing to its great convenience. For example, it generally requires a single plasmid carrying a mini-CRISPR and a donor template to achieve precise gene editing or a single plasmid carrying a mini-CRISPR and a small *acr* gene to achieve targeted gene regulation. In addition to the convenience for endogenous genetic applications, type I systems also display several other superiorities in microbial gene editing and regulation when they are compared to the class 2 Cas9 or Cas12a systems. For instance, they showed higher efficiency in genome targeting and editing as well as lower cytotoxicity in some strains. In addition, type I systems possess a greater capacity to generate deletions of large genomic fragments. Moreover, in some clinical and environmental non-model and genetically recalcitrant microbial species such as *S. acidocaldarius*, which grows at a temperature of 75 °C and pH of 3 [74], type I systems that are endogenously encoded in these species might be preferred or the only choice to facilitate functional genomics.

## 8. Challenges and future perspectives

Despite the apparent convenience and high efficiency of gene editing and gene regulation using type I systems, it is worth noting that most developed type I CRISPR-Cas gene-editing systems work only in particular strains, i.e., their hosts. In addition, more than half of bacterial strains do not carry endogenous CRISPR-Cas systems, and some of the systems may be inactive due to the absence or natural mutation of some Cas subunits or the presence of type-specific Acr proteins encoded from the genome [17]. Introducing a different type I system to these hosts for heterologous gene editing is expected but remains a bottleneck due to the challenge of delivering and expressing multiple Cas proteins. As demonstrated, integrating the large *cas* operon into the genome of target hosts will facilitate stable expression of type I systems and then the use of these systems for gene editing and gene regulation. By overcoming the delivery and expression issues other than Tn7 or other genome integrative strategies that are only applicable in some Gram-negative bacterial strains, type I systems will become more useful as universal tools for genome editing and gene regulation in microorganisms.

It has been mentioned that type I systems are naturally present in many clinically, environmentally and industrially important microbial strains or can be delivered to different strains. However, gene editing based on these systems remains difficult in most hosts, frequently owing to the extremely poor capacity of DNA recombination. Due to the lack of available gene-editing tools in these strains, deleting or mutating Cas3 nuclease genes in type I systems to alternatively repurpose them as CRISPR-Cas-based gene-regulation tools for functional characterizations of genes of interest is also difficult. Therefore, prevention of Cas3-mediated genome cleavage without the deletion or mutation of this gene in the genome is important to achieve gene repression in these strains. Acr proteins exhibit distinct structures and mechanisms to inactivate the CRISPR-Cas immune function [105]. In addition to AcrIE1, AcrIF3 and AcrIF23, accumulating Acrs from different subtypes were identified to specifically prevent the recruitment of Cas3 nucleases to the targeting site or inhibit their nuclease activities. These Acrs are promising to reconstitute the abundant endogenous type I systems as endogenous CRISPRi systems. Moreover, as different lengths of spacers in the crRNA enable the CRISPR-Cas system to execute different functions of target DNA binding or cleavage, optimizing the spacer length to avoid DNA cleavage is another strategy to realize gene regulation. Thus, simultaneous expression of crRNA and Acrs or the optimization of spacer length could be considered to promisingly achieve type I CRISPRi for the investigations of fundamental biological questions or metabolic engineering in genetically recalcitrant microbial strains.

## Use of AI tools declaration

The authors declare they have not used artificial intelligence (AI) tools in the creation of this article.
